# COVID-19 and the Efficacy of Different Types of Respiratory Protective Equipment Used by Health Care Providers in a Health Care Setting

**DOI:** 10.7759/cureus.7621

**Published:** 2020-04-10

**Authors:** Talia Malik

**Affiliations:** 1 Internal Medicine, Wah Medical College, Wah Cantonment, PAK

**Keywords:** covid-19, health care outcomes, n95

## Abstract

Coronavirus, the virus that caused the global pandemic at the beginning of 2020 and affected millions across the globe, presented as an enormous challenge to health care providers around the world. With increasing numbers of infected patients presenting daily, health care workers are struggling to take effective measures to protect themselves from transmission against the highly contagious coronavirus. This case helps us understand the implications of coronavirus-infected patients on the health care providers directly responsible for the management of these patients and the relative efficacy of different types of respiratory protective equipment mainly N95 masks and surgical masks in preventing the spread of infection among those at the front lines providing care.

## Introduction

The coronavirus (COVID-19) was declared a global pandemic by World Health Organization (WHO) on March 11, 2020 after it was known to originate from Wuhan, China, and resulted in more than 381,000 confirmed cases around the world at the time [1,2]. Very little is known about its effect among those responsible for its management and treatment, primarily the doctors, nurses, and the first responders. The coronavirus causes an acute respiratory infection that is transmitted by contact and droplet routes. The use of personnel protective equipment (PPE), such as surgical gloves, face masks, eye protection, and regular hand washing, is known to limit the spread of the virus, but little is known about the relative efficacy of N95 masks over the regular surgical masks in preventing the transmission of the virus among health care workers (HCWs). This aspect of identifying the superior type of respiratory protective equipment (RPE) is of significant importance in order to provide maximal protection to HCWs, prevent medical equipment shortages, and to lessen the burden on manufacturers and suppliers of this equipment. Both types of face masks are known to prevent the transmission of respiratory particles; however, the N95 masks are thought to have a superior efficacy by filtering out very small particles. In spite of having many benefits, the N95 masks have certain limitations and it is important to understand if the benefits outweigh the risks when compared to surgical masks. This case helps us better understand the efficacy and benefits of different types of RPE used by HCWs during the management of patients infected with the coronavirus.

## Case presentation

A 55-year-old woman with a history of diabetes mellitus was admitted to the intensive care unit (ICU) in early March 2020 for severe shortness of breath. She had recently returned from a trip to Iran and was suspected to have been in contact with people infected with the coronavirus. On admission, she was given high-flow supplemental oxygen that slightly improved her shortness of breath; however, after a few hours, her respiratory status worsened and she was intubated and mechanically ventilated in the ICU. The HCWs who performed the aforementioned procedures used either the N95 mask or the surgical mask depending upon availability and personal preference. The patient’s respiratory status gradually improved over a period of two days. In accordance with existing guidelines, the patient was extubated and shifted to the isolation ward for further management. Three samples of nasopharyngeal swabs were obtained from the patient for three consecutive days for COVID-19 testing and all came out positive for severe acute respiratory syndrome coronavirus 2 (SARS-Cov-2) on polymerase chain reaction (PCR) assay. All the 34 HCWs exposed to the patient were isolated and quarantined for a period of 14 days. Information was obtained about the type of RPE used by each HCW during exposure to the patient. Half of the HCWs used surgical masks, whereas the rest used N95 masks as the RPE while performing procedures on the infected patient. During this period of quarantine, routine surveillance monitoring for cough, shortness of breath, and muscle aches was made. Two samples of nasopharyngeal swabs were obtained from the HCWs on the day of exposure and the last day of quarantine for COVID 19 testing via the PCR assay. Each HCW remained asymptomatic and tested negative for COVID 19 on both tests. 

## Discussion

The coronavirus is known to spread through aerosolized particles. HCWs are at an increased risk of being infected without proper PPE and protective measures. This case highlighted the importance of evaluating the efficacy of different RPE during a viral pandemic in protecting the HCWs from the transmission of infection. None of the HCWs who were exposed to the virus tested positive for COVID-19 despite using the different types of RPE. This helps us in determining the relative efficacy of surgical masks and N95 masks over one another in preventing the transmission of the virus among HCWs. It can also be deduced that both types of RPE offer equal protection to HCWs from the virus; however, these findings need to be validated by well-designed large-scale studies. The observation made in this case report was also highlighted by a previous study which showed that N95 masks were not superior to surgical masks for preventing influenza infection among HCWs [3]. Another study concluded that there was no significant difference between N95 mask and surgical masks in preventing the risk of transmission of respiratory infections from infected patients [4]. Although the above studies showed no significant difference in efficacy of both types of RPE, it is vital to understand that different infectious agents have different mechanisms of transmission and action, and hence large-scale randomized controlled trials (RCTs) need to be conducted to better understand the particular pattern and characteristics of SARS-Cov2 that differentiate it from other respiratory infectious agents with similar features. The N95 mask is thought to be superior to the surgical masks. These masks are known to filter out 95% of small airborne particles including bacteria and viruses. They have been tested and approved by the National Institute of Occupational Safety (NIOS); however, there are certain limitations to its use. Since breathing while wearing the N95 mask is harder, it is not recommended for the elderly, claustrophobics, and individuals suffering from lung diseases as it may exacerbate their pre-existing conditions. These masks need to be properly fitted on the face every time to ensure that a proper seal is in place to provide maximal protection. The inability to form a proper seal does not provide adequate protection. This can be difficult to achieve in individuals with facial hair and in children. It can also inadvertently lead to frequent contact between the hands and the face while adjusting the mask, which further increases the risk of transmission. The tight seal also leads to the build-up of heat and humidity within the mask causing discomfort and difficulty in breathing. The duration and cost of manufacturing are longer and higher and during a pandemic, shortages of this equipment can result in avoidable exposure to HCWs. During a pandemic or health crisis, HCWs need to be able to access PPE readily in order to protect themselves, patients, and their contacts. Interpretation of this case and the above studies helps us understand the need to urgently conduct large-scale RCTs and incorporate the findings of these trials and studies to revise the existing guidelines regarding the use of RPE by HCWs and possibly increase the usage of readily available surgical masks in favor of N95 masks for preventing COVID-19 transmission. Till the availability of any conclusive evidence, health care providers in direct contact with COVID-19 cases should continue the use of N95 respirators as advocated by the current guidelines. 

## Conclusions

Although this case report helps us in determining the relative efficacy of different RPE in preventing COVID-19 transmission, there is still a lot more that needs to be studied about the transmission and pathology of the coronavirus to introduce effective measures and equipment that will protect HCWs in the future. The intricate details about the size and transmission of the virus still need to be studied in depth to figure out the best method of RPE that can be used to prevent its transmission. Although this case report signifies that there is no superior protection offered by N95 masks in comparison to surgical masks, it has certain limitations and additional studies, particularly RCTs need to be conducted in a health care setting to determine the effectiveness of different RPE, which may lead to the revision of existing policies and guidelines regarding the best ways to protect HCWs from being infected with the coronavirus in the event of an exposure.
